# The theoretical calculation with laparoscopic and 3D-MSCT validation of the volume measurement method for a gastric pouch in gastric bypass surgery

**DOI:** 10.3389/fsurg.2026.1708352

**Published:** 2026-03-25

**Authors:** Oral Ospanov, Kassymkhan Sultanov

**Affiliations:** 1 Department of Surgical Diseases and Bariatric Surgery of Astana Medical University, Astana, Kazakhstan; 2 Surgery Center of Professor Oral Ospanov, Astana, Kazakhstan

**Keywords:** calculation, gastric bypass, gastric pouch, laparoscopic, validation, volume measurement

## Abstract

The authors proposed a method for calculating gastric pouch volume based on its geometric shape, validated using visual laparoscopic and 3D-MSCT measurements. The method simplifies the calculation of the volume of the created gastric reservoir. This method can be reproduced and used in further studies on gastric pouch measurement.

## 1 Introduction

Obesity is a chronic, multifactorial disease and represents one of the most pressing global health challenges of the twenty-first century, affecting over 1 billion people worldwide (1). There has been a sharp increase in the prevalence of this disease (2).

Gastric bypass remains a reliable method in metabolic and bariatric surgery. Mechanical restriction of food volume by reducing the size of the gastric pouch is important to weight loss outcomes after gastric bypass. Improved weight loss outcomes have been demonstrated after gastric pouch reduction procedures performed as a secondary procedure to stop weight gain (3). Volumetric assessment of gastric pouches using multi slice computed tomography (MSCT) enables important pathological measurements to be made and provides useful information for the selection of appropriate revision surgery after bariatric surgery (4, 5).

The gastric pouch is typically formed using a linear stapler, which ensures that the organ walls are sutured in the desired shape and size. We have not found any information in the literature on how theoretical calculations are used in the formation and linear measurement of the gastric pouch. The gastric pouch is usually cut out with the help of a cylindrical gastric bougie. Therefore, we hypothesize that a formula to calculate the volume of a geometric cylinder that accurately conveys the shape of the gastric pouch can be used to determine the volume of the gastric pouch. However, we have not found a simple method of visual laparoscopic assessment in the literature to confirm the accuracy of the theoretical calculations.

The aim of our study was to use a mathematic formula to determine the volume of the cylinder and calculate the volume of the gastric pouch to provide practical validation of this new method for measuring the volume of the gastric pouch.

## 2 The theoretical measuring method

**Figure 1:**
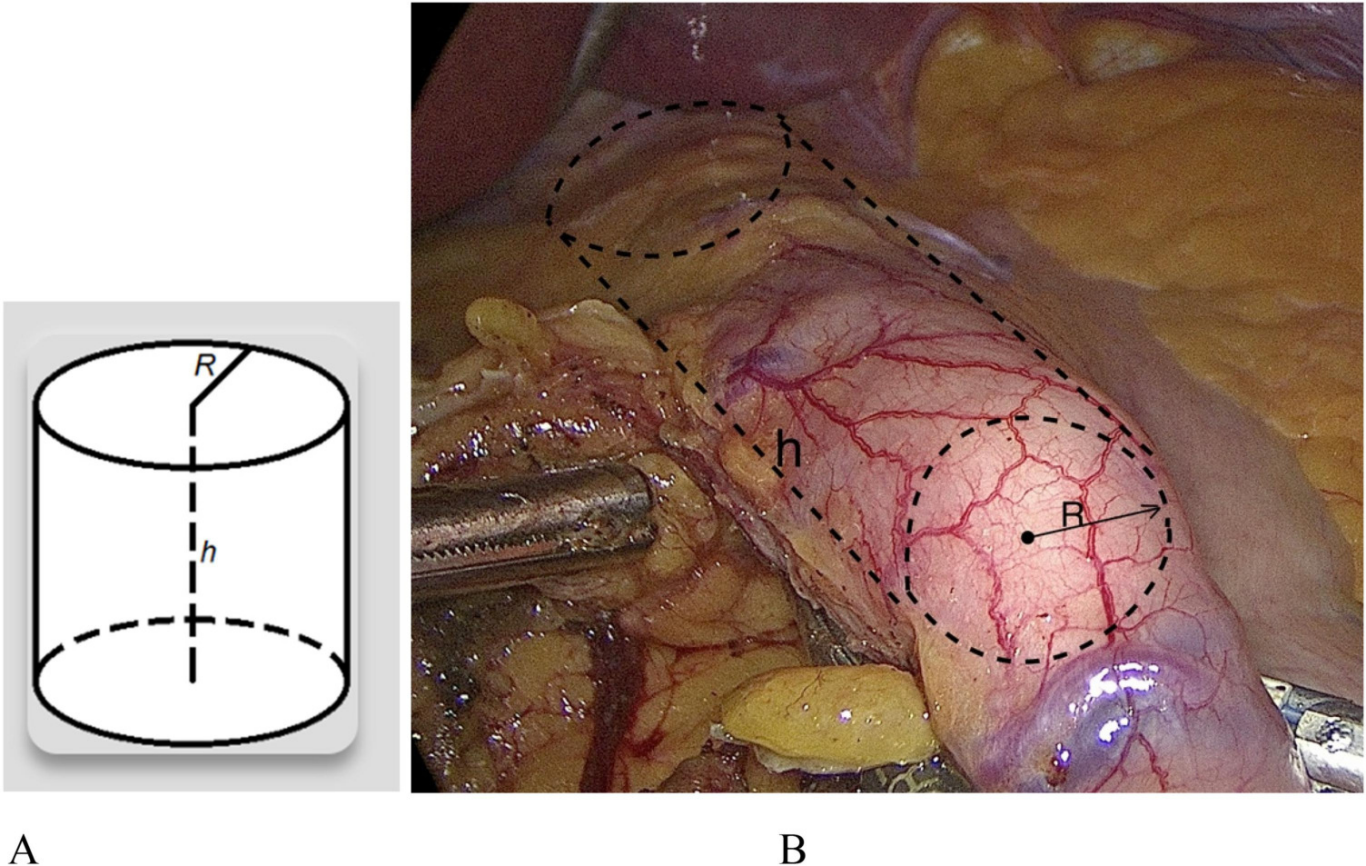
**(A)** measurement of the volume of the gastric pouch. Formula for the cylinder for calculation of gastric pouch volume: (V = π × R^2^ × h): π = 3.14, where R is the radius of the gastric pouch, h is the height (long gastric pouch), and x is the multiplication sign. **(B)** The contours of the intragastric bougie (tube) are visible against the background of an anatomical whole stomach (before transaction), and lines (black color) simulating the shape of a cylinder have been added.

Using the formula to determine the volume of the cylinder (Figure 1A), we calculated the volume of the gastric pouch (Figure 1B) (6).

## 3 The results of the calculation of the volume of the gastric pouch

In our calculations, we used the diameter value of the applied gastric tube Fr/mm: 32 Fr. This is 10.7 mm in metric measurement. We added the indent size from the edge of the gastric tube and staple line to this value, approximately +5 mm. As a result, the inner diameter of the gastric pouch was 15.7 mm. To apply the formula, we found out the radius from the last diameter result (R = 7.85 mm). The calculation result is demonstrated in Table 1. A measure of volume in the metric system of the gastric pouch uses the formula for the cylinder.

**Table 1:** Calculation of the volume of the gastric pouch.

Height (length) of the gastric pouch (h)	Diameter of the gastric calibration tube: 32 Fr (D1)	Distance between the edge of the gastric tube and the stapler line/internal diameter (radius) of the gastric pouch + D1/D2 (R)	Volume of gastric pouch (V = π × R^2^ × h), π = 3.14 R is the radius of the gastric pouch; h is the height (long gastric pouch); x is the multiplication sign (cc).
100 mm	10.7мм	+5 мм/ D2 = 15.7 mm (R = 7.85 mm)	Calculation by formula V = 3,14 × 616,225 × 100 = 19,359,279 mm^3^ (≅20 ml)

As shown in Table 1, including the measurement results in the calculation formula, we obtained the gastric pouch size. The capacity of the gastric pouch was calculated to be ≅20 ml.

## 4 Practical method for validating gastric pouch volume

### 4.1 Conditions and preparation of the gastric pouch for validation of the theoretical calculation

**Figure 2:**
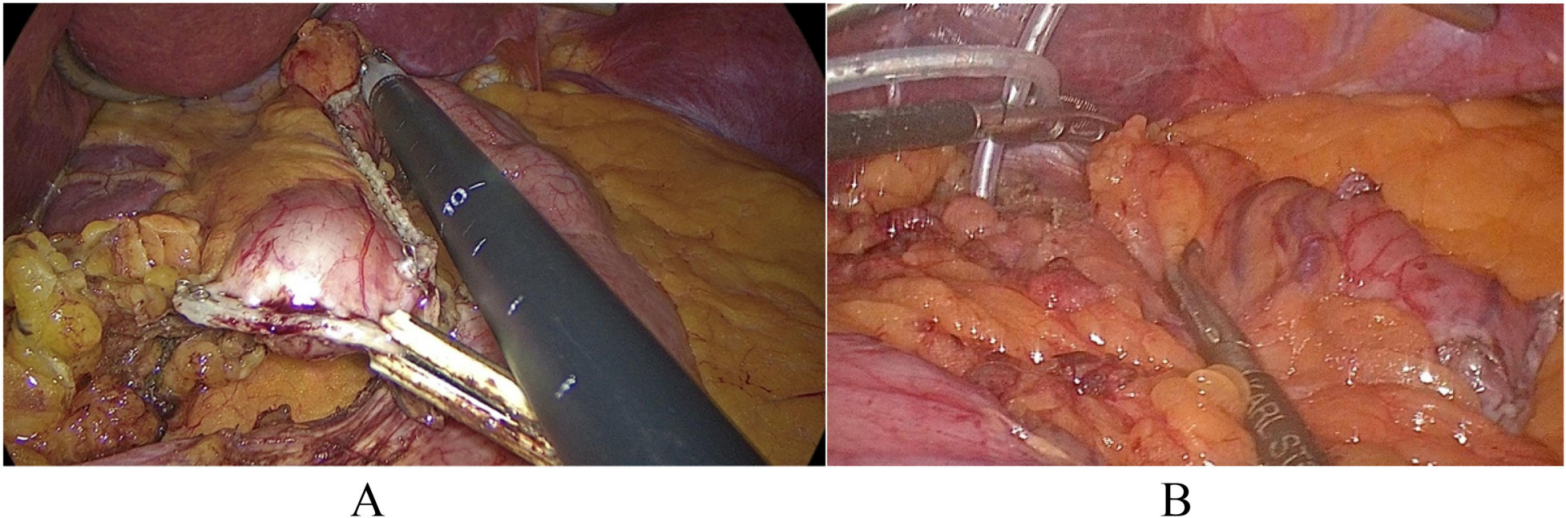
**(A)** gastric pouch created after transection of the stomach transversely to the axis of the stomach and crossing the stomach longitudinally along the gastric bougie. The transverse stapler line is captured with a white clamp. The longitudinal stapler line was measured using black instruments (L = 10 cm).The gastric bougie in the cavity of the gastric pouch is pulled proximally to the esophagogastric junction. **(B)** The tourniquet is left on the abdominal part of the esophagus (above the proximal end of the gastric pouch).

The measurement is carried out after the creation of the gastric pouch without performing a gastroenteroanastomosis (Figure 2A). The stomach cavity is open only from the esophagus side.

A 32 Fr. size bougie (tube) is inserted into the esophagus.

A retroesophageal tunnel is formed behind the abdominal portion of the esophagus. A soft silicone tube is passed through this posterior esophageal tunnel around the esophageal bougie and the esophageal walls at the level of the gastroesophageal junction (across proximal end of gastric pouch). Both ends of the silicone tube are connected in rings using tourniquet-type clamps to seal the gastric pouch cavity at the top (Figure 2B). Since the gastric pouch remains blind (closed) at the end of the future gastrojejunostomy, sealing the distal end of the gastric pouch is not required.

Using a gripper, the compression of the esophagus walls is adjusted on the esophageal bougie located inside. The tourniquet ensures tightness between the esophagus walls and the bougie when introducing fluid into the gastric pouch.

**Figure 3:**
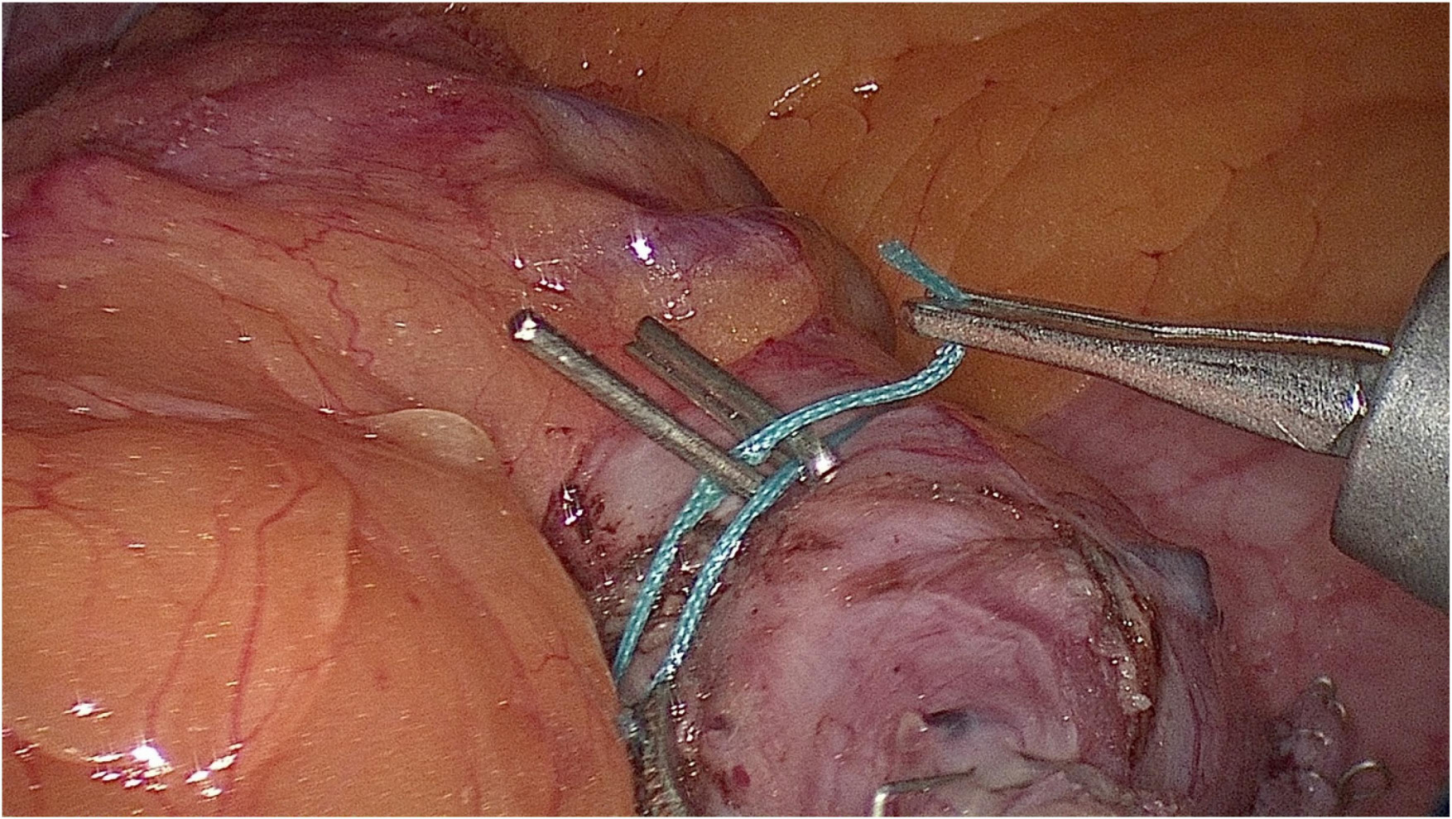
Two clips clamped on a thread are placed next to each other until (before) the cavity of the gastric pouch is filled with liquid.

On the part of the stomach free of venous vessels, a thread is placed circularly in a transverse arrangement of two clips located next to and touching each other (Figure 3).

### 4.2 Measurement capability

From the oral end of the bougie, the fluid obtained by theoretical calculation using the formula for determining the volume of a cylinder is introduced.

The rate of introduction is 1 ml per 1 s.

**Figure 4:**
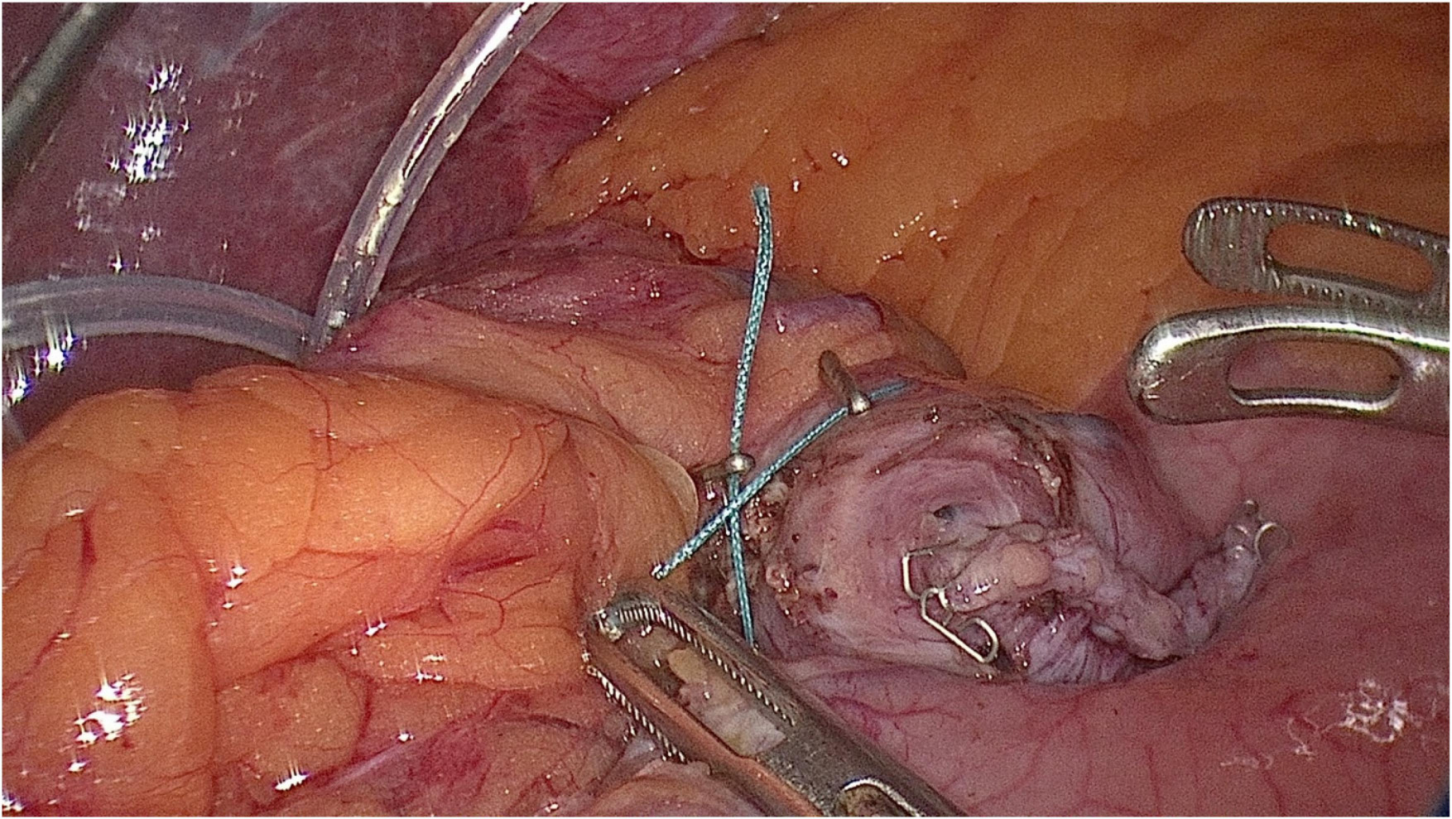
The moment of divergence of the clamps after filling the cavity of the gastric pouch through a nasoesophageal bougie with 20 ml liquid.

The result is obtained as follows: after a continuous diptych with the amount of fluid introduced (ml) into the gastric pouch, the moment of divergence of the clips is determined; this will be considered the actual volume of the gastric pouch (Figure 4).

The obtained result is compared with the carefully calculated volume of the pouch's stomach.

We compared the volume (≅20 ml^3^) obtained in the theoretical calculation with the minimum volume of fluid introduced into the gastric pouch, at which there was no divergence of the two clips (marker); this practical result (indicator) exactly corresponded to the theoretical calculation. Further introduction of fluid led to divergence of the two clips, which indicated the expansion of the gastric pouch by more than 20 ml^3^.

For comparison, we used 3D-MSCT, which is more modern than conventional CT. The gastric pouch volumetry protocol was similar to that described by Ayuso, S.A. et al. (7). To prevent stretching of the gastric pouch, the study was conducted during morning fasting and for 2–3 days after surgery.

Table 2 compares the validation results of the developed method against three MSCT scans in a case series. As can be seen in Table 2, the theoretical calculation was always equal to 20 ml, but in practical laparoscopic validation it was in the range from 18 to 22 ml and on average amounted to19.6 ± 1.4 ml. In 3D-MSCT gastric pouch volumetry, the range was from 16 to 26 ml, and the mean value was 21.7 ± 3.9 ml. The absence of statistical significance of differences (*p* = 0.17) in different validation groups indicates the same accuracy of volume measurements.

**Table 2:** Validation of the theoretical calculation of the volume of the gastric pouch.

*N* patients	Pouch volume (ml)			*P*-value
	Theoretical calculation (ml)	Practical laparoscopic validation (ml)	3D-MSCT gastric pouch volumetry (ml)	
1	20	19	24	–
2	20	22	16	–
3	20	20	22	–
4	20	19	26	–
5	20	18	19	–
6	20	20	22	–
7	20	21	18	–
8	20	18	27	–
Mean ± Std Dev		19.6 ± 1.4	21.7 ± 3.9	0.17 (NS)

## 5 Discussion

Routine use of CT volumetric analysis to measure gastric pouch size is important to study the relationship between pouch size and incidence of ulcer formation (7, 8). Various CT volumetric methods provide accurate data on the volume of the gastric pouch. However, for bariatric and metabolic surgeons it is important to have an alternative validated method for theoretical calculation of the volume of the gastric pouch when planning surgical interventions.

We propose using fundamental mathematical data to calculate the volume of the gastric pouch. We also describe a simple and accessible method for checking theoretical calculations in the conditions of a laparoscopic procedure.

The results of the validation of the theoretical calculation of the volume of the gastric pouch (19.6 ± 1.4 ml (laparoscopic validation) vs. 21.7 ± 3.9 ml (volumetry of the gastric pouch using 3D-MSCT)) showed there were no statistically significant differences between the validation groups (*p* = 0.168).

Based on the favorable results of our previous studies, we believe that a gastric pouch volume of 20 ml is optimal (9–12). It is important to note that the theoretical calculation is correct for gastric pouches that utilize gastric bougies on the unstretched walls of the hollow organ. The presence of the bougie during calibration transfers the volume of the bougie to the volume of the gastric pouch. But further introduction of liquid can lead to a sharp increase in the volume of the gastric pouch due to the elasticity of the walls of the hollow organ. To validate the theoretical calculations, we propose using the visual laparoscopic method. This method can be reproduced and used in further studies.

Before our proposal, gastric pouches were cut in a wide range of lengths, resulting in gastric pouch volumes that ranged from 20 ml to 60 cc (ml) (13–15). As can be seen from the literature, creating a gastric pouch with a threefold difference in gastric pouch volume is, unfortunately, a common practice among surgeons. This creates issues for researchers who unsuccessfully try to study the outcomes of bariatric surgery using gastric pouch sizes in meta-analyses. Due to the variability of data, this is difficult because there are no requirements for standardized sizes and no simple preoperative calculation method that utilizes metric data. We use a standardized approach to create gastric pouches (16, 17). The accuracy of the gastric pouch volume is important for comparing results and determining the dependence of results, for example, weight loss on the gastric pouch volume. Therefore, our method allows us to avoid gross inaccuracies in gastric pouch creation. Volumetric assessment of gastric pouches using multi-slice computed tomography (MSCT) can only be applied after the creation of a gastric pouch, but our method can be used in the preoperative period to accurately plan the length and width of the gastric pouch and select the diameter of the gastric bougie.

To prevent gastric pouch stretching, the study of MSCT volumetric assessment of gastric pouches was conducted within 2–3 days after surgery because, according to endoscopic ultrasound, the volume of the gastric pouch increases over the course of a year. The use of gastric bypass with fundoplication (FundoRing) showed less gastric pouch stretching in the FundoRing-OAGB group (44.39 ± 7.60 ml) compared with the OAGB group (50.44 ± 12.01 ml), where a conventional gastric pouch was used. By the third year, volume expansion continued (FundoRing-OAGB volume 60.39 ± 10.04 ml vs. 87.93 ± 25.79 ml in the OAGB group) (18).

### 5.1 Limitations of the study

The obtained measurement results apply only to the edged gastric pouch, without stretching its walls. The deformation parameters of the gastric pouch were not considered in the calculations because one of the conditions for the proper creation of a gastric pouch is the creation of the correct cylinder shape during surgical dissection of the gastric pouch from a normal (intact primary anatomical) stomach. With proper cutting, there is no deformation of the walls due to the calibration of the gastric pouch dimensions on a tubular gastric bougie, which serves as a strong frame during the creation of a new anatomical model with a geometric cylinder shape, and the frame eliminates deformation of the stomach walls.

## 6 Conclusion

Our theoretical calculation for measuring the gastric pouch volume in gastric bypass surgery has been validated by practical laparoscopic methods and 3D-MSCT. Our method can be applied in metabolic bariatric surgery for anatomical models with a geometric cylinder shape. The absence of statistically significant differences in different validation groups indicates accurate volume measurements.

## Data Availability

The original contributions presented in the study are included in the article/Supplementary Material, further inquiries can be directed to the corresponding author.
